# Resistance to Bleomycin-Induced Lung Fibrosis in MMP-8 Deficient Mice Is Mediated by Interleukin-10

**DOI:** 10.1371/journal.pone.0013242

**Published:** 2010-10-07

**Authors:** Emilio García-Prieto, Adrián González-López, Sandra Cabrera, Aurora Astudillo, Ana Gutiérrez-Fernández, Miriam Fanjul-Fernandez, Estefanía Batalla-Solís, Xose S. Puente, Antonio Fueyo, Carlos López-Otín, Guillermo M. Albaiceta

**Affiliations:** 1 Department of Biología Funcional, Universidad de Oviedo, Instituto Universitario de Oncología (IUOPA), Oviedo, Spain; 2 Department of Bioquímica y Biología Molecular, Universidad de Oviedo, Instituto Universitario de Oncología (IUOPA), Oviedo, Spain; 3 Department of Cirugía y Especialidades Médicoquirurgica, Universidad de Oviedo, Instituto Universitario de Oncología (IUOPA), Oviedo, Spain; 4 Unidad de Cuidados Intensivos, Hospital Universitario Central de Asturias, CIBER Enfermedades Respiratorias-Instituto de Salud Carlos III, Oviedo, Spain; McMaster University, Canada

## Abstract

**Background:**

Matrix metalloproteinases (MMPs) may have pro and antifibrotic roles within the lungs, due to its ability to modulate collagen turnover and immune mediators. MMP-8 is a collagenase that also cleaves a number of cytokines and chemokines.

**Methodology and Principal Findings:**

To evaluate its relevance in lung fibrosis, wildtype and *Mmp8^−/−^* mice were treated with either intratracheal bleomycin or saline, and lungs were harvested at different time points. Fibrosis, collagen, collagenases, gelatinases, TGFβ and IL-10 were measured in lung tissue. *Mmp8^−/−^* mice developed less fibrosis than their wildtype counterparts. This was related to an increase in lung inflammatory cells, MMP-9 and IL-10 levels in these mutant animals. *In vitro* experiments showed that MMP-8 cleaves murine and human IL-10, and tissue from knockout animals showed decreased IL-10 processing. Additionally, lung fibroblasts from these mice were cultured in the presence of bleomycin and collagen, IL-10 and STAT3 activation (downstream signal in response to IL-10) measured by western blotting. In cell cultures, bleomycin increased collagen synthesis only in wildtype mice. Fibroblasts from knockout mice did not show increased collagen synthesis, but increased levels of unprocessed IL-10 and STAT3 phosphorylation. Blockade of IL-10 reverted this phenotype, increasing collagen in cultures.

**Conclusions:**

According to these results, we conclude that the absence of MMP-8 has an antifibrotic effect by increasing IL-10 and propose that this metalloprotease could be a relevant modulator of IL-10 metabolism *in vivo*.

## Introduction

The accumulation of collagen fibers in the lung interstitium is a form of abnormal repair in some respiratory diseases. The primary forms of this syndrome are largely unknown and grouped under the term idiopathic pulmonary fibrosis [1]. Lung fibrosis can be also secondary to different types of injury, such as persistent acute respiratory distress syndrome, asbestos or silica exposure or treatment with drugs such as bleomycin [2]. Chronic inflammation is one of the mechanisms leading to these diseases. This is especially relevant in secondary fibrosis, but its involvement in the idiopathic forms is discussed [3]. The clinical course of this pathology shows a poor response to different therapies including steroids and other immuno-suppressors, and often evolves to irreversible respiratory failure [4].

Matrix metalloproteinases (MMPs) are a family of enzymes involved in different processes such as modulation of inflammation, tissue remodeling and collagen processing [5]. These enzymes play an important role in the pathogenesis of pulmonary fibrosis [6]. By targeting different substrates and being controlled by diverse regulatory mechanisms [7], MMPs establish a complex network in which different enzymes may play opposite roles. Likewise, specific family members may also play different roles in different time points of the disease. This is the case for MMP-8, also known as collagenase-2. This enzyme can digest native collagen, but its function in vivo seems to be more related to the control of the inflammatory response [8], [9]. By the ability to cleave different cytokines and chemokines, MMP-8 promotes the initial onset and the later clearance of the neutrophilic inflammatory response. Mice lacking MMP-8 show a delayed wound healing due to a persistent inflammatory infiltrate [10]. Additionally, it has been observed a decreased collagen deposition in the wounds of these animals. This abnormal response could be of interest within the lungs challenged with a profibrotic stimulus. Therefore, we hypothesized that mice lacking MMP-8 would develop less lung fibrosis than their respective wildtype counterparts. To test this hypothesis, we used a widely known model of lung fibrosis based on the administration of intratracheal bleomycin [11]. This drug induces an acute inflammatory response followed by the development of fibrosis, allowing to study the putative role of MMP-8 by assessing the differences between wildtype and *Mmp8* knockout mice after injury.

## Results

### Lung fibrosis is decreased in Mmp8^−/−^ mice

**Figure 1:**
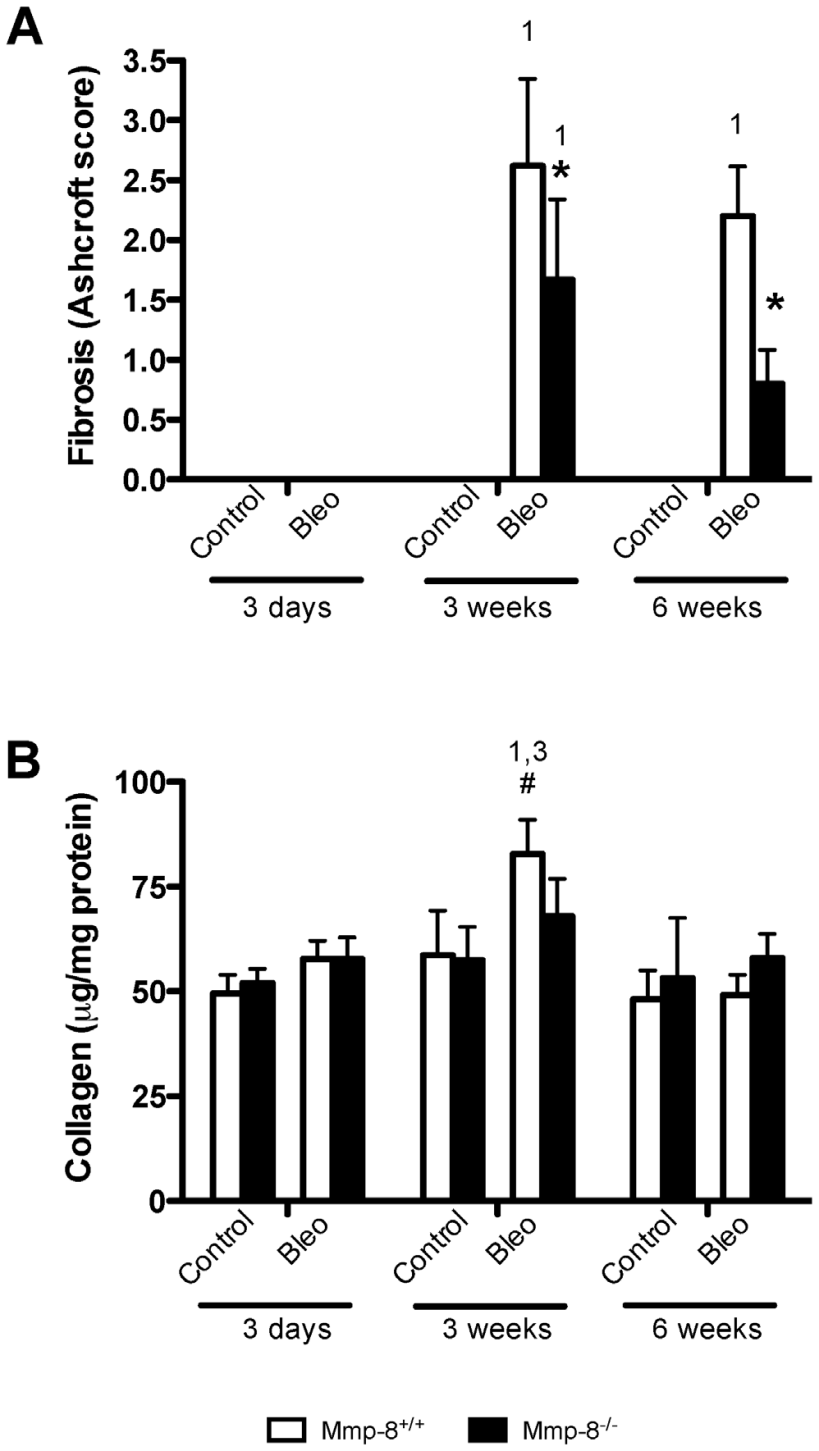
Decreased fibrosis in mice lacking MMP-8 challenged with intratracheal bleomycin. Fibrosis was quantified using the Ashcroft score (**A**) and recently synthesized collagen (**B**) measured in lung homogenates (n≥6 per group). ^1,2,3^P<0.05 in post-hoc test when compared against 3 days (1), 3 weeks (2) or 6 weeks (3) within the same genotype and treatment; *p<0.05 when compared against wildtype within the same time and treatment; ^#^p<0.05 when compared against saline within the same time and genotype.

**Figure 2:**
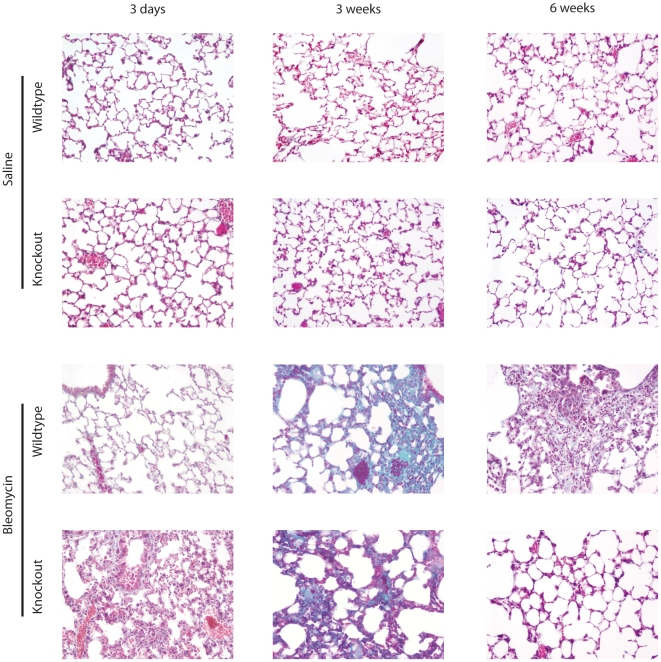
Representative histological sections (20×) from each experimental group. After staining with Masson's trichrome, fibrosis appears as green fibers, whereas epithelial and inflammatory cells are stained in red.

Lung fibrosis was quantified using the Ashcroft scale in histological sections stained with Masson's trichrome (Figure 1A). No sign of fibrosis was detected in saline-treated mice of either genotype. Bleomycin injection induced a fibrotic response after 3 weeks. However, the degree of fibrosis was lower in MMP-8 deficient animals, as mutant mice scores were significantly lower than their wildtype counterparts (p<0.05 in post-hoc pairwise comparisons). After 6 weeks, fibrosis persisted in wildtype mice, but was partially solved in mutant animals. Consistent with this finding, there was a peak in recently synthesized collagen (Figure 1B) only in wildtype mice 3 weeks after bleomycin injection. Knockout mice showed a non-significant increase in this parameter (Figure 1B). Representative histological preparations are shown in Figure 2.

Bronchiolization appeared only after bleomycin administration, and not in saline-treated mice and increased progressively up to 6 weeks after injury. There were no differences between genotypes (data not shown).

### Changes in collagenolytic and gelationlytic activities after bleomycin treatment

**Figure 3:**
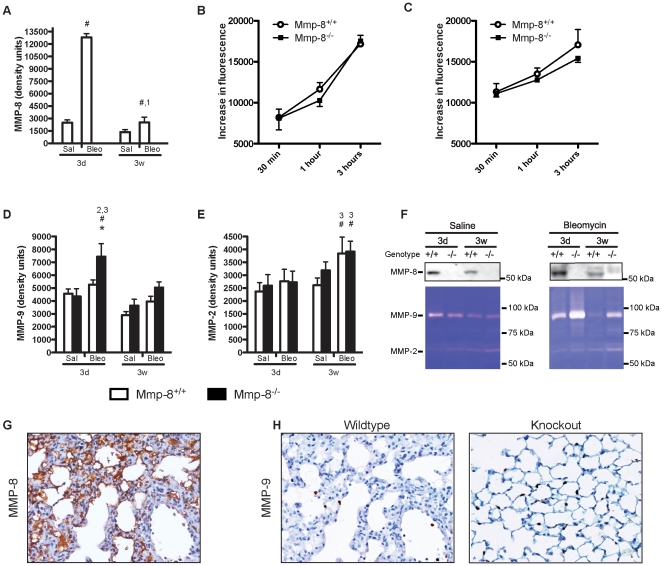
MMP activity in each experimental group. **A**: MMP-8 increased after bleomycin instillation. As expected, no MMP-8 was detected in knockout mice (n≥7 per group). **B–C**: Collagenolytic activity of lung tissue homogenates from wildtype and knockout mice 3 days (B) and 3 weeks (C) after bleomycin instillation (n = 3 per group). There are no significant differences between genotypes. **D**: MMP-9 has an acute increase after bleomycin instillation only in knockout mice (n≥7 per group). **E**: MMP-2 increases in both wildtype and knockout mice 3 weeks after injury (n≥7 per group). **F**: Representative western blotting and gelatin zymography used to quantify these MMPs. G–H: Immunohistochemical staining of MMP-8 (G) and MMP-9 (H) after bleomycin instillation. ^1,2,3^p<0.05 in post-hoc test when compared against 3 days (1), 3 weeks (2), 6 weeks (3) within the same genotype and treatment; *p<0.05 when compared against wildtype within the same time and treatment; ^#^p<0.05 when compared against saline within the same time and genotype.

Collagenases and gelatinases act sequentially to degrade collagen. To study the implication of MMP-8 in the pathogenesis of bleomycin-induced fibrosis we measured its level in lung homogenates (Figure 3A). This enzyme increased 3 days after bleomycin instillation, to decrease later. Of note, saline-treated mice showed a modest increase in this enzyme, suggesting that the instillation procedure can induce a minor inflammatory response within the lungs. As expected, MMP-8 was not detected in knockout mice.

Then, we focused on differences in other collagenases and gelatinases to discard compensatory mechanisms in mutant mice. First, we assessed collagenolytic activity of lung homogenates (3 days and 3 weeks after bleomycin treatment). There were no differences in total collagenolytic activity between wildtype and knockout mice, thus discarding an overcompensatory increase of other collagenolytic enzymes in *Mmp8^−/−^* mice that could explain the decreased fibrosis (Figure 3B–C).

Regarding gelatinases, knockout mice showed a significant increase in MMP-9 in lung homogenates when studied 3 days after bleomycin instillation (Figure 3D). Values returned to baseline levels at the other time points of the study. In contrast, wildtype mice showed only a small increase that did not reach statistical significance. MMP-2 (Figure 3D) is another gelatinase involved in the pathogenesis of lung fibrosis [12]. We observed an increase in this enzyme in both wildtype and knockout mice 3 weeks after injury. There were no changes in any gelatinase in saline-treated animals. MMP-8 (Figure 3G) was expressed mainly in fibroblasts, whereas MMP-9 expression was restricted to inflammatory cells, mainly neutrophils, as shown by immunohistochemistry (Figure 3H).

### Increased inflammatory infiltrate in Mmp8^−/−^ mice

**Figure 4:**
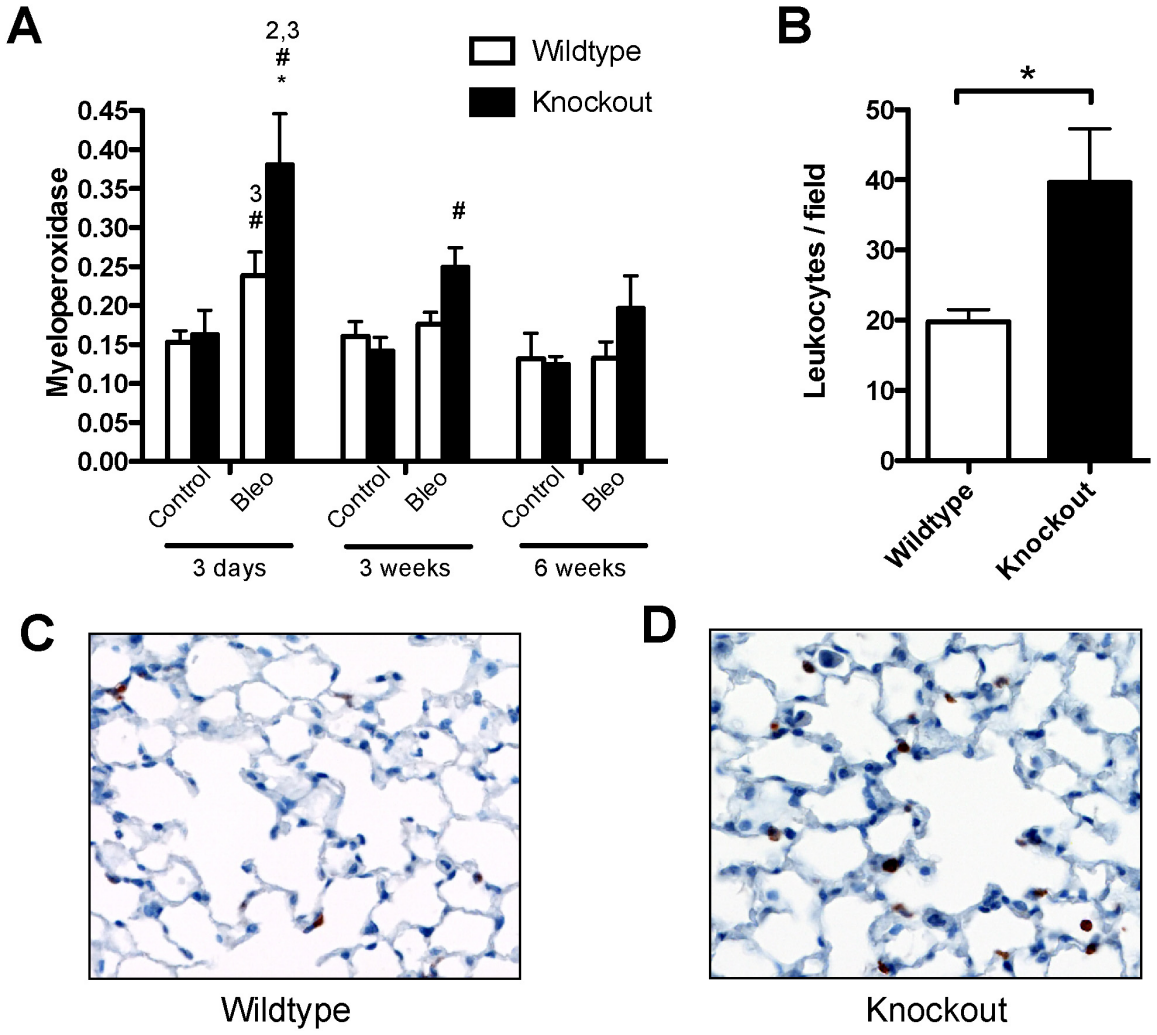
Increased inflammatory infiltrate activity in *Mmp8^−/−^* mice treated with bleomycin. **A**: Myeloperoxidase was considered a surrogate marker of neutrophilic infiltration of lung tissue (n≥7 per group). **B**: The number of myeloperoxidase-positive cells was higher in knockout mice. **C–D**: Representative immunohistochemistry for myeloperoxidase in wildtype (C) and knockout (D) animals. ^1,2,3^P<0.05 in post-hoc test when compared against 3 days (1), 3 weeks (2), 6 weeks (3) within the same genotype and treatment; *p<0.05 when compared against wildtype within the same time and treatment; ^#^p<0.05 when compared against saline within the same time and genotype.

Bleomycin causes an acute inflammatory response within the lungs, which was measured by quantifying myeloperoxidase activity in tissue extracts. Myeloperoxidase (Figure 4A) increased in both wildtype and knockout mice 3 days after bleomycin injection, but not after saline. Noteworthy, that increase was more pronounced in knockout mice, as demonstrated by immunohistochemical analysis showing higher counts of myeloperoxidase-positive cells (Figure 4B–D). In mutant animals, increased levels of myeloperoxidase persisted along the 6 weeks of the experiment, whereas returned to baseline levels in wildtype mice.

### Th2 cytokines TGFβ and IL-10 are differentially regulated in Mmp8^−/−^ mice

**Figure 5:**
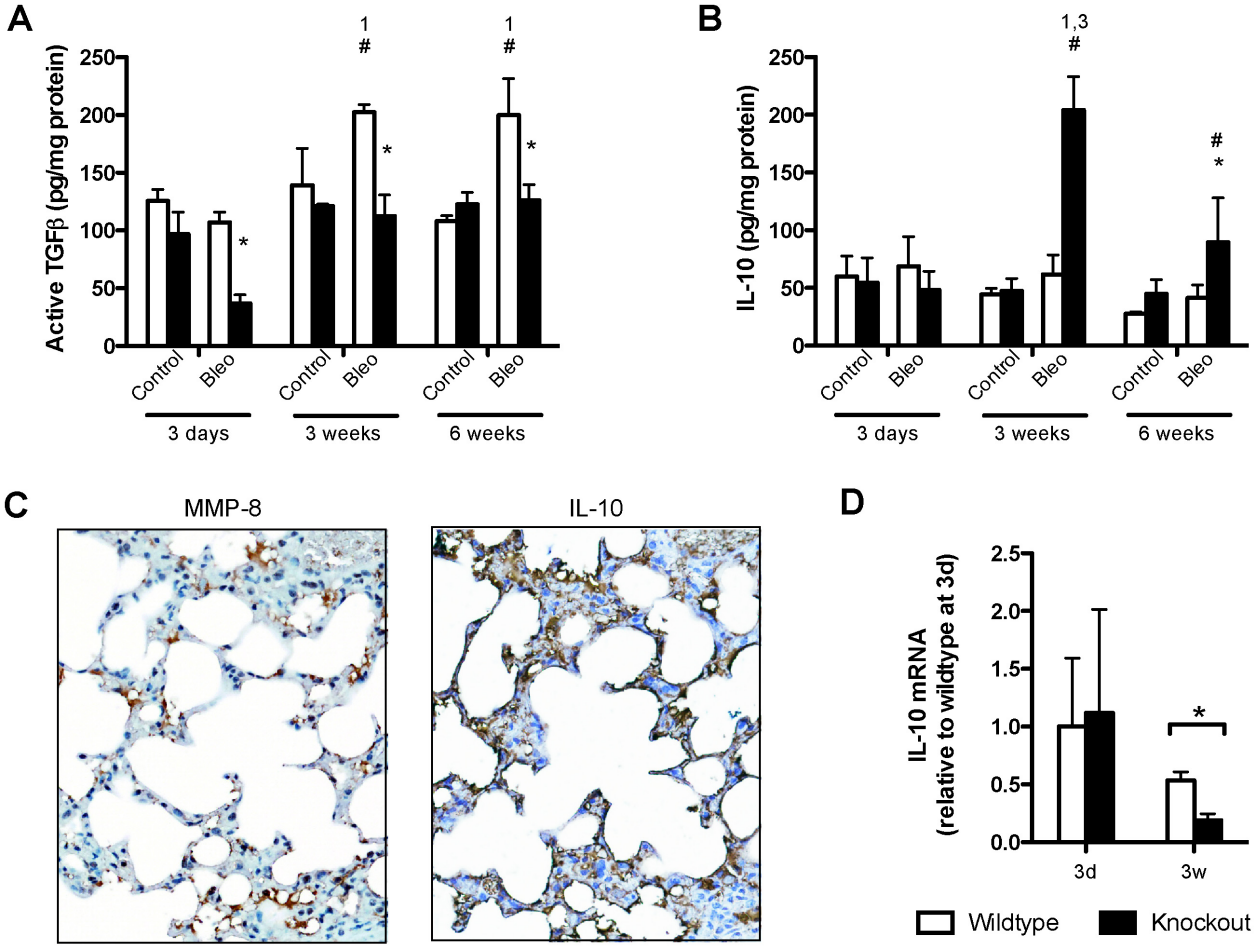
Cytokines in lung homogenates. **A**: TGFβ increases after bleomycin-induced lung injury only in wildtype mice, whereas knockout mice show lower level in all the stages of the experiment. **B**: IL-10 increases in knockout mice during the chronic phase. N≥5 per group. **C**: Immunohistochemistry for IL-10 and MMP-8 in lungs from wildtype mice after bleomycin instillation, showing IL-10 expression in epithelium and fibroblasts within the alveolar septa. MMP-8 was expressed in fibroblasts. D: IL-10 expression after bleomycin instillation shows no differences between genotypes after 3 days, and a significant decrease in IL-10 mRNA in knockout mice 3 weeks after instillation. ^1,2,3^P<0.05 in post-hoc test when compared against 3 days (1), 3 weeks (2), 6 weeks (3) within the same genotype and treatment; *p<0.05 when compared against wildtype within the same time and treatment; ^#^p<0.05 when compared against saline within the same time and genotype.

Th2 cytokines could play a relevant role in the development of fibrosis after bleomycin instillation. TGFβ is a profibrogenic factor that increased 3 and 6 weeks after bleomycin treatment only in wildtype mice. In contrast, *Mmp8*-deficient mice showed lower levels of this cytokine in the early phase (3 days) and values comparable to saline-treated mice at the other time points (Figure 5A).

IL-10 is another anti-inflammatory cytokine that can suppress TGFβ synthesis, thus exerting antifibrotic effects. There were no changes in this cytokine in saline-treated mice of both genotypes, or in bleomycin-treated wildtype animals. However, *Mmp8^−/−^* mice showed a 3-fold increase in IL-10 3 weeks after bleomycin treatment, and a smaller but still significant increase persisted 6 weeks after injury (Figure 5B). Immunohistochemical studies demonstrated IL-10 expression in epithelial cells and fibroblasts, whereas MMP-8 was expressed in fibroblasts (Figure 5C). When IL-10 gene expression was studied, mRNA levels of this cytokine in baseline conditions were undetectable. However, they were detected 3 days after bleomycin instillation with no differences between genotypes. Interestingly, IL-10 mRNA decreased significantly in knockout mice 3 weeks after bleomycin (Figure 5D). These findings open the possibility that IL-10 could be responsible for the benefits seen in terms of fibrosis in knockout mice and suggests that MMP-8 can be a relevant modulator of IL-10 function.

### MMP-8 processes IL-10 *in vitro* and *in vivo*

**Figure 6:**
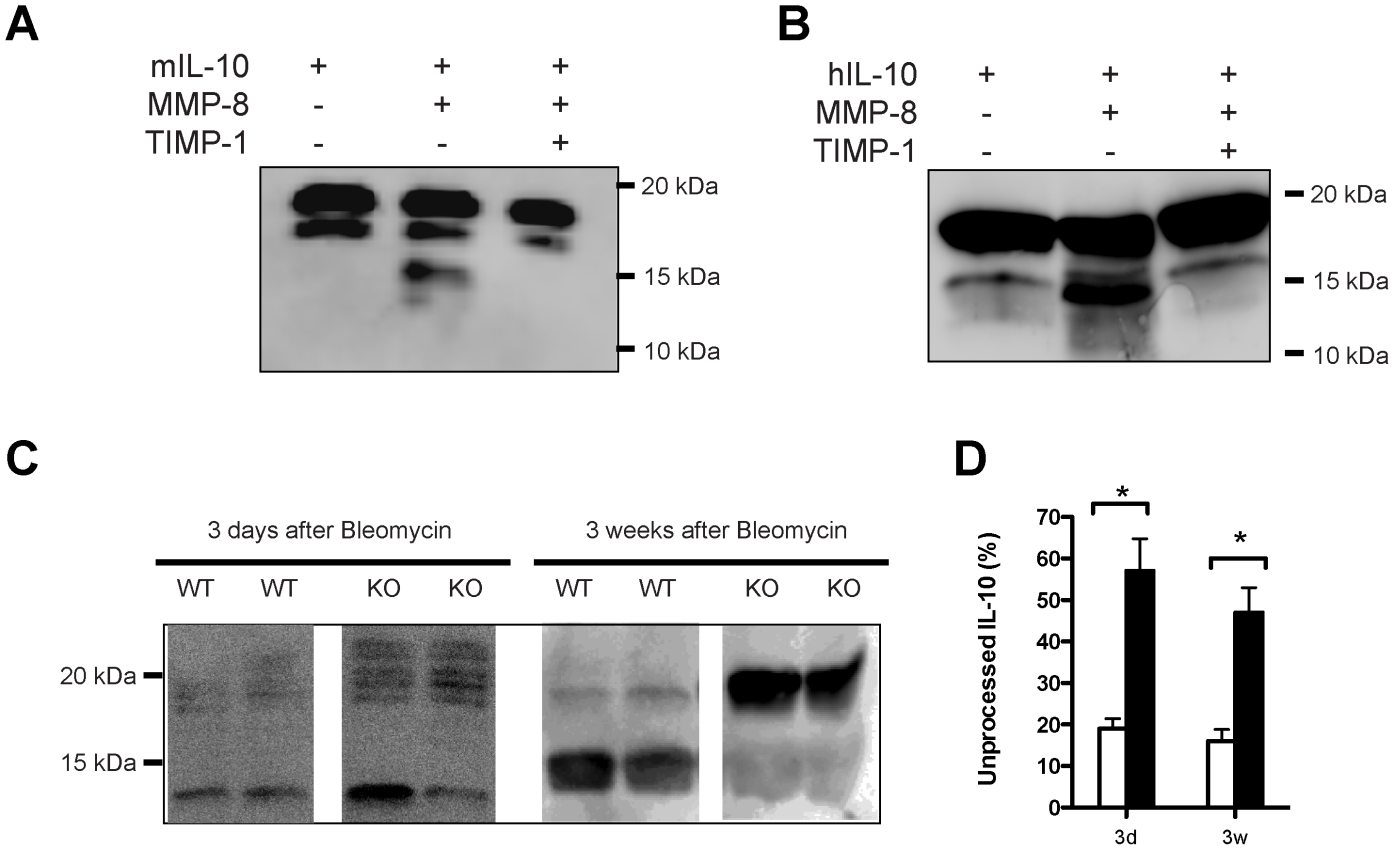
*In vitro* cleavage of IL-10. MMP-8 processing of murine (**A**) and human (**B**) IL-10 in vitro. Cleavage results in a 14 kDa fragment in addition to intact IL-10 (18 kDa). In vivo cleavage (**C**) also occurs. Although there is a 14 kDa fragment in both genotypes, indicating overlapping of other proteases, the percentage of intact IL-10 is significantly higher (*p<0.05) in knockout mice (**D**), demonstrating the relevance of MMP-8 in IL-10 metabolism in vivo.

To explore the possibility that IL-10 could be a substrate of MMP-8, recombinant murine IL-10 was incubated with this enzyme. After 18 hours of incubation, proteolytic processing of the cytokine was demonstrated by western blotting, showing that IL-10 (18 kDa) was converted to lower molecular mass species (∼14 kDa). The appearance of these species was inhibited by TIMP-1 (Figure 6A). We repeated this experiment using recombinant human IL-10, showing the same result (Figure 6B).

In vivo processing of IL-10 by MMP-8 was studied in lung tissue homogenates by western blotting using a polyclonal antibody against mouse IL-10. Two IL-10 positive bands were detected at 18 and 14 kDa, corresponding to intact and processed IL-10 (Figure 6C). The percentage of intact IL-10 was higher in knockout mice three days and three weeks after bleomycin instillation (Figure 6D), demonstrating that absence of MMP-8 results in higher levels of unprocessed IL-10 and, therefore, that MMP-8 is an important regulator of IL-10 function in vivo.

### Antifibrotic response in Mmp8^−/−^ fibroblasts is IL-10 dependent

**Figure 7:**
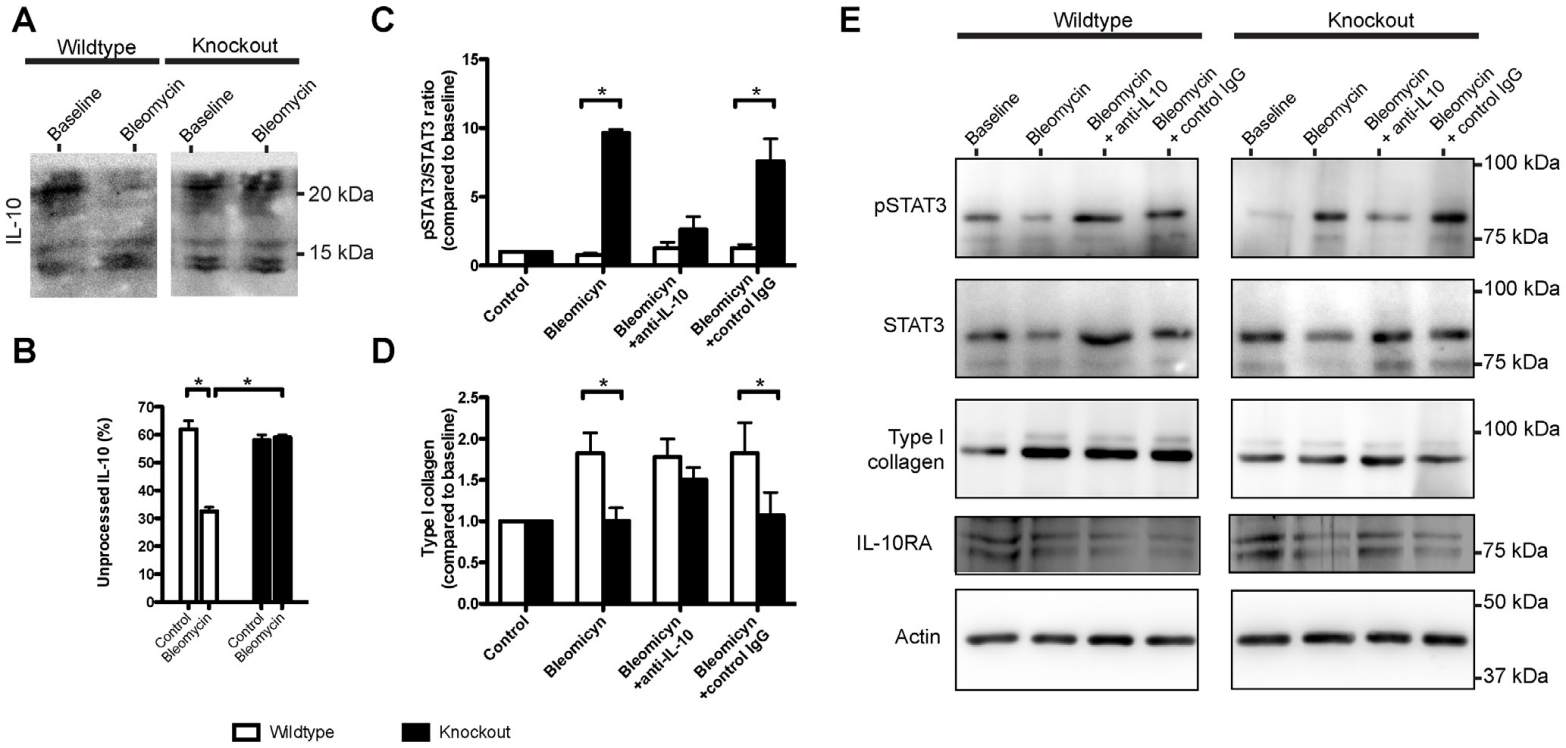
Biological activity of IL-10. The effects of MMP-8 on IL-10 activity were studied in cultured lung fibroblasts from wildtype and knockout mice (n = 4 per group). Intact IL-10 decreased in wildtype cells cultured in presence of bleomycin (Representative western blot: **A**; quantification: **B**). This was related to a decrease in phosphorylated STAT3 (**C**), demonstrating a decrease in the activity of IL-10 signaling pathway, and an increase in Type I collagen (**D**). In contrast, fibroblasts from knockout mice showed no IL-10 cleavage (**A–B**), an increase in STAT3 phosphorylation (**C**) and decreased collagen synthesis (**D**). Addition of a neutralizing antibody against IL-10 decreased STAT3 activation and increased collagen. Addition of a control IgG had no effects. Panel **E** shows representative western blots of these experiments. *p<0.05 compared against wildtype under the same culture conditions.

The relationship between IL-10 processing and lung fibrosis suggested by the above experiments was demonstrated in a culture model. Lung fibroblasts from wildtype and mutant mice were treated with bleomycin. After bleomycin challenge, there was an increase in IL-10 processing in wildtype fibroblasts, and intact IL-10 (18 kD) was significantly decreased. However, there was no change in IL-10 processing in cultures from knockout mice (Figure 7A–B). We also studied STAT3 phosphorylation as a marker of IL-10 activity. The ratio of phosphorylated to non-phosphorylated STAT3 was markedly increased in cultures from knockout mice in the presence of bleomycin. This increase was significantly attenuated when a blocking anti-IL10 antibody was added to the medium, thus demonstrating that it was caused by an increase in bioactive IL-10 (Figure 7C) and therefore suggesting that the cleaved form of IL-10 has a decreased activity. In agreement with the results observed in mice, collagen increased after bleomycin treatment in cell cultures from *Mmp8^+/+^* mice but not from *Mmp8^−/−^* animals. The addition of an IL-10 blocking antibody increased collagen levels in cultures from *Mmp8^−/−^* mice up to those seen in their wildtype counterparts (Figure 7D). These results demonstrate that the decreased fibrosis seen in mutant mice is caused directly by the increase in IL-10. There were no differences in IL-10 receptor expression (Figure 7E). Representative western blots of these experiments are shown in Figure 7E.

## Discussion

Our results demonstrate that absence of MMP-8 results in an anti-fibrotic effect after bleomycin administration. This effect is mediated by the absence of proteolytic cleavage and subsequent inactivation of endogenous IL-10. Collectively, these results demonstrate that MMP-8 is an important regulator of IL-10 in vivo and point this metalloprotease as a potential therapeutic target in lung fibrosis.

Matrix metalloproteinases have been involved in different phenomena that occur during inflammation and tissue repair, such as modulation of the immune response, extracellular matrix turnover or cell migration [13], [14]. There is clear evidence that MMPs play a key role in lung fibrosis [6], [15], although different enzymes may have opposite effects. For instance, it has been documented that mice lacking MMP-7 are protected from bleomycin-induced fibrosis [16]. In contrast, overexpression of MMP-9 protects against lung fibrosis [17] and absence of this gelatinase resulted in decreased bronchiolization but no differences in fibrosis [18]. This variety of effects highlights the need of specific inhibitors for a clinical application of the experimental findings [19].

The decreased collagen levels in mice lacking a collagenase suggested that the benefits seen in these animals were not related to the effects of MMP-8 on the extracellular matrix proteins. Moreover, our results show no overcompensatory increase in other collagenases, and even a slightly decreased collagenolytic activity in knockout mice. As the Sircol assay only measures newly synthesized collagen, and not covalent cross-linked fibers, the lower levels of collagen seen in mutant animals suggest a decreased rate of synthesis. This can also explain the absence of differences in soluble collagen seen after 6-weeks in spite of a marked difference in histological scores.

Therefore, we focused on mechanisms other than collagen degradation. MMP-8 has some interesting effects in acute and chronic inflammation. Animals lacking this enzyme have a delayed onset of the acute inflammatory response in different experimental models [8], [10], [20]. However, once established, the inflammatory infiltrate persists for longer times than in controls. Moreover, MMP-8 may also regulate lung permeability [21], which is an important determinant of outcome in pulmonary fibrosis [22], and apoptosis of inflammatory cells [23]. It has been proposed that MMP-8 is a master regulator of neutrophil chemotaxis by cleavage of chemokines such as MIP-1α [24] or LIX [9], the murine ortologue of human IL-8. This increased inflammation after bleomycin instillation has been recently described in detail [24]. Based on these results, the decreased fibrosis seen in or mice cannot be explained by lower levels of acute damage, as lung injury at 7 days after bleomycin instillation is even higher in mutant animals.

A collateral effect of this delayed clearance of inflammation in a model of skin wounds was a decreased collagen deposition and therefore a delayed healing [10]. Our results follow the same pattern, but in this case the decreased fibrosis is beneficial rather than a side effect. In contrast to the findings in MMP-9 deficient mice [18], we did not observe differences in bronchiolization.

Many of the results seen in disease models using mice lacking MMPs are due to differences in cytokine and chemokine processing. Cleavage of these immune mediators by MMPs can modulate their activity [25]. Different cytokines are involved in the pathogenesis of pulmonary fibrosis. TGFβ is the most important profibrotic mediator [26]. This cytokine has also an anti-inflammatory effect. Of note, the pattern of increased inflammation and decreased fibrosis after bleomycin instillation has been observed in mice lacking the integrin α_v_β_6_, which is an activator of latent TGFβ [27].

Another cytokine involved in the regulation of the inflammatory response, fibrosis and TGFβ regulation is IL-10. We found an increase in this cytokine in mice lacking MMP-8 submitted to ventilator-induced lung injury [28]. Moreover, it has been published that interleukin-10 can exert antifibrotic effects after bleomycin instillation. Arai and coworkers [29] demonstrated that overexpression of IL-10 reduced fibrosis in vivo. Similar results were found by Nakagome et al [30], who showed in bleomycin-injected mice that IL-10 decreased TGFβ expression in the lung, especially in macrophages. Part of the inhibitory effect of IL-10 on TGFβ is caused by a decreased expression of integrin α_v_β_6_. However, this cannot be the only mechanism responsible for decreased TGF in knockout mice, as these animals show lower levels even before the increase in IL-10 (i.e. 3 days after bleomycin). Although it has been reported that *Mmp8^−/−^* mice have a defective TGFβ signaling pathway, the underlying mechanisms are not completely elucidated [10].

Similar results were found using a model of fibrosis associated with chronically inhaled endotoxin [31]. However, there are also reports of a profibrotic role of IL-10 in lung fibrosis induced by silica [32]. Differences in injury model could have been responsible for the discrepancies.

**Figure 8:**
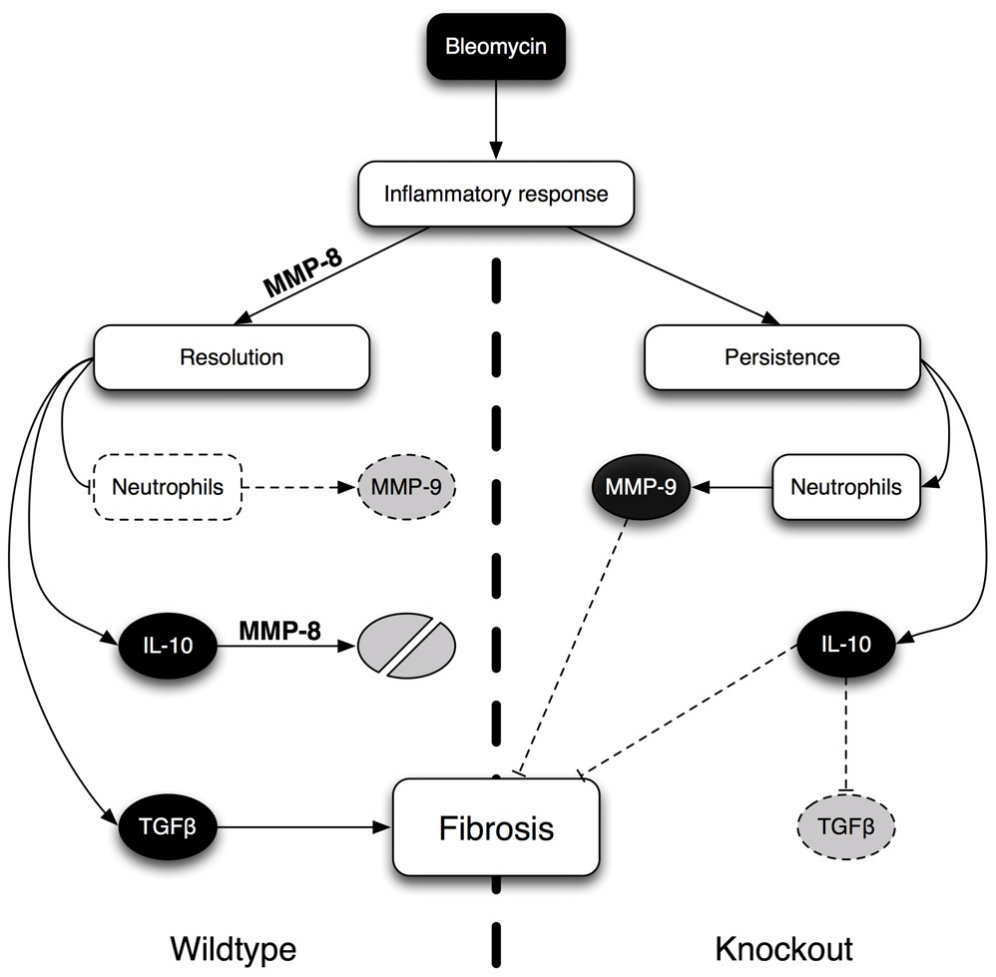
Schematic representation of the mechanisms by which absence of MMP-8 ameliorates lung fibrosis. In normal mice (left), presence of MMP-8 facilitates the resolution of inflammation, thus decreasing MMP-9 (by the clearance of neutrophils) and IL-10 (by cleavage). The increased TGFβ promotes fibrosis. In absence of MMP-8 (right), there are increased levels of MMP-9 and IL-10, both of them with antifibrotic properties. IL-10 can also inhibit TGFβ synthesis. For further details, see text.

We have shown here that IL-10 is inactivated by MMP-8. This result is reinforced by the absence of differences in IL-10 expression, and even by the decrease in IL-10 expression coinciding with the peak of protein levels. It has been published that IL-10 exerts a paracrine negative feedback involving its own receptors [33]. Moreover, the culture model suggests that the antifibrotic effect seen in our mutant mice is IL-10-dependent. Using IL-10 deficient animals, Kradin and coworkers have not found a difference in lung fibrosis [34]. This is in agreement with our findings, as one can expect that released IL-10 in wildtype mice is inactivated by MMP-8. Collectively, our results are consistent with a model in which absence of MMP-8 results in increased inflammation, but also increased levels of IL-10. Recruited neutrophils release MMP-9, which has a protective role in bleomycin-induced fibrosis [17] through cleavage of potential profibrotic mediators such as insulin-like growth factor binding protein-3 (IGFBP3). Simultaneously, intact IL-10, either directly or by decreasing TGFβ [30], ameliorates the fibrotic process, therefore leading to the beneficial effects seen in knockout mice. This mechanism is depicted in figure 8.

Based on these findings, MMP-8 could be a therapeutic target in lung fibrosis. For this strategy to be effective, selective inhibitors should be used to avoid interference with other MMPs with antifibrotic properties. However, this therapeutic approach should be viewed with caution by different reasons: First, the increased inflammatory response seen in absence of MMP-8 could be detrimental and promote additional injury. Although there was no increased lung damage in *Mmp8^−/−^* mice, and the role of inflammation in idiopathic fibrosis is discussed, this could be a relevant side effect. Second, the bleomycin model is not fully representative of the human disease, neither idiopathic nor secondary [11].

Another implication of our results is that MMP-8 is an important regulator if IL-10 activity in vivo. This anti-inflammatory cytokine has been proposed as a therapeutic agent in inflammatory and autoimmune diseases [35]. In these cases, the possibility of targeting MMP-8 to increase the levels of active IL-10 could be considered.

In conclusion, we have demonstrated that absence of MMP-8 has an antifibrotic effect mainly driven by an increase in IL-10. Therefore, selective inhibition of this enzyme could be a therapeutic approach in pulmonary fibrosis. Our findings illustrate how this MMP plays a key role in the regulation of the inflammatory response and the subsequent repair. Moreover, they open the possibility to modulate IL-10 metabolism by MMP-8 targeting in a variety of diseases.

## Methods

### Animals

Eight-week-old male wildtype and *Mmp8^−/−^* mice generated in a mixed C57/Sv129 background [8] were used. Genotypes were confirmed by PCR. The Animal Research Committee of the Universidad de Oviedo authorized the experimental protocol.

### Experimental design

Mice of both genotypes were randomly assigned to bleomycin or saline administration. Under anesthesia using vaporized fluorane, a midline neck incision was done and the trachea exposed. Either bleomycin (2 units/Kg) or isotonic saline were administered intratracheally using a 28G needle. After this, the skin was sutured and the animals left to recover. Four mice (1 saline-treated *Mmp8^−/−^*, 1 bleomycin-treated *Mmp8^−/−^* and 2 bleomycin-treated *Mmp8^+/+^*) died within the first 5 days after instillation and were replaced. There were no deaths after this time point. Mice were sacrificed 3 days, 3 weeks or 6 weeks after the intratracheal instillation. This results in 12 different groups, depending on the genotype, treatment and time course after the injury. We included 8-10 animals per group (total 106 mice).

### Histological analysis

The left lung was fixated using formaldehyde. Three lung slices, stained with Masson's trichrome, were studied. Lung fibrosis was scored from 0 (no fibrosis) to 8 (massive fibrosis in all fields) using the Ashcroft scale [36]. Bronchiolozation (presence of bronchial-like cells in the alveoli) was quantified using the Jensen-Taubman scale [37]. A pathologist (AA), blinded to genotypes and experimental conditions, made all the evaluations.

To evaluate the inflammatory infiltrate within the lungs, immunohistochemical analysis was done in sections from bleomycin-treated mice of both genotypes using an anti-myeloperoxidase antibody (Thermo Fisher Scientific, USA). The number of positive cells in three microscopic fields (×20) was counted and averaged. Immunostaining in lung sections was done using antibodies against MMP-8 and IL-10 (Abcam, UK) and MMP-9 (Santa Cruz Biotechnologies, USA).

### Collagen content

Soluble lung collagen was measured in lung homogenates using the Sircol assay (Biocolor, UK), following manufacturer's instructions. Briefly, lung tissue homogenates were mixed with the Sircol dye and centrifugated. The pellet was resuspended in NaOH and the optical density at 540 nm measured. The obtained absorbance is proportional to the abundance of recently synthesized collagen.

### Quantification of MMP-8 by western blot

The levels of MMP-8 in lung tissue were measured by western blotting. Lung homogenates were resolved in a 8% SDS-polyacrilamide gel, transferred to a nitrocellulose membrane and incubated with a polyclonal antibody raised in rabbits against murine MMP-8. A secondary peroxidase-linked anti-rabbit antibody was used to detect MMP-8 by chemoluminescence, as previously described. Membranes were scanned in a LAS-3000 camera (Fujifilm, Germany), and intensity of the bands measured using the ImageJ software.

### Collagenolytic activity

Total collagenolytic activity was quantified in lung homogenates using the EnzChek collagenase assay kit (Invitrogen, USA). Briefly, fluorescein-conjugate collagen (DQ collagen, Invitrogen, USA) and tissue homogenates (100 micrograms of total protein) were incubated at 37°C in a reaction buffer. The increase in fluorescence, measured at 0.5, 1, 3 and 5 hours, is proportional to collagenolytic activity of the tissue.

### Gelatin zymography

MMP-2 and MMP-9 were measured in lung homogenates using gelatin zymography. Protein content was adjusted to 20 micrograms in all samples, and loaded in a SDS-polyacrylamide gel containing gelatin, electrophoresed and incubated overnight in an buffer (150mM NaCl, 5mM CaCl2, 50mM Tris-HCl, pH 7.6). After staining with Coomassie Blue, gelatinolytic activity appears as white bands in a blue background. The gels were scanned and quantified using ImageJ software (National Institutes of Health, USA).

### Myeloperoxidase assay

Myeloperoxidase activity was measured as a marker of neutrophil infiltration. A lung fragment was homogenated in phosphate buffer containing CTAB. Tissue homogenates were incubated with O-dianisidine and H_2_O_2_ and light absorbance at 460 nm was measured as a marker of myeloperoxidase content.

### Cytokine measurements

Total IL-10 and active TGFβ were measured in lung homogenates using commercial ELISA kits (eBioscience, UK), following manufacturers' instructions. Additionally, IL-10 expression was measured by quantitative PCR. Total lung RNA was extracted from mouse lungs using TRIzol reagent (Invitrogen Life Technologies,USA) and reversed transcribed into cDNA (Advantage RT-for-PCR Kit; Clontech, USA). Quantitative reverse transcription-PCR was carried out in duplicate for each sample using 20 ng of cDNA, TaqMan Universal PCR master mix and 1 µL of the specific TaqMan custom gene expression assay for IL10 (Mm01288386_m1, Applied Biosystems, USA). To quantify gene expression, PCR was performed at 95°C for 10 min, followed by 40 cycles at 95°C for 15 s, 60°C for 30 s, and 72°C for 30 s using an ABI Prism 7700 sequence detector system. Gene expression was normalized to β-actin as control. Relative expression of the analyzed genes was calculated according to manufacturer's instructions.

### IL-10 digestion

To assess enzymatic processing of IL-10 by MMP-8, murine or human IL-10 were incubated with MMP-8 pre-activated with APMA, at 37°C in a buffer containing 150 mM NaCl, 5 mM CaCl2, 50 mM Tris-HCl (pH 7.6). Experiments without MMP-8 or adding TIMP-1 to the incubation buffer were conducted in parallel. After incubation, the samples were resolved by 15% SDS-PAGE. The proteins were transferred to a nitrocellulose membrane and detected by western blotting as described before, using polyclonal anti-mouse or anti-human IL-10 antibodies (purchased from Abcam, UK and R&D Systems, USA, respectively). To clarify the *in vivo* relevance of the cleavage of IL-10 by MMP-8, we detected the cytokine in lung tissue homogenates from wildtype and knockout mice 3 weeks after bleomycin administration. IL-10 was detected by western blotting using the same polyclonal antibody after 15% SDS-PAGE. Bands of different molecular weight were detected, the intensity of each one was quantified using ImageJ software. The percentage of unprocessed IL-10 with respect to the total amount was computed for each sample.

### IL-10 activity assay in cell cultures

Fibroblast cultures were initiated from lung explants from WT and MMP8 deficient mice as described [38]. Lungs were washed with PBS, and triturated with razor blades. The disrupted explants were allowed to adhere to the bottom of the culture plates before covering them with DMEM cell culture medium supplemented with penicillin/streptomycin/gentamicin, 10% FBS, nonessential amino acids (0.1 mM), sodium pyruvate (1 mM), L-glutamine (2 mM) and N-2-hydroxyethylpiperazine-N′-2-ethanesulfonic acid (HEPES) (10 mM). Cultures were maintained at 37°C and 5% CO_2_. After the first 2 weeks of culture, most nonfibroblast cells died while the fibroblasts established as the predominant cell type. Then, cultured cells were trypsinized, filtered, and washed. Fibroblasts were seeded in 12-well plates with DMEM without FBS under the following conditions: Baseline, bleomycin (bleomycin 100 nM), bleomycin plus IL-10 blockade (bleomycin 100 nM and anti-IL10 blocking antibody purchased from Peprotech Inc) and bleomycin plus unspecific IgG (Abcam, UK). After 12 hours, supernatants were removed and cell extracts obtained using a RIPA buffer (100 mM TRIS pH 7.4, 150 mM NaCl, 10 mM EDTA, 1% deoxycolic acid, 1% Triton X-100, 0.1% SDS, 50 mM NaF, 1 mM orthovanadate and protease inhibitor cocktail) and stored at −80°C. In these extracts, western blots were done to measure type I collagen (using an antibody purchased from Calbiochem, Merck, UK), IL-10 and IL-10 receptor (IL-10RA antibody, Abcam, UK). To measure IL-10 activity, we quantified the phosphorylation balance of the transcription factor STAT-3 by western blot using antibodies against phosphorylated and non-phosphorylated forms of STAT3 (Signalway Antibody Co, USA). As loading control we used a polyclonal anti-actin antibody purchased from Santa Cruz Biotechnology Inc (USA). Detection and quantification of the bands was done as previously described.

### Statistical analysis

Results are expressed as mean±SEM. Variables were compared using an ANOVA, including genotype, treatment and time of study as factors. When appropriate, post-hoc tests were done using Bonferroni's correction. Comparisons between two variables were done using a T test. A p value lower than 0.05 was considered significant.
